# Site-Directed Immobilization of DuraPETase onto PET-Binding PDA@SiO_2_ for High-Efficiency PET Degradation

**DOI:** 10.3390/molecules31101675

**Published:** 2026-05-15

**Authors:** Zixuan Li, Fengyuan Zhang, Shaolei Zhao, Mingbo Sun, Jingru Liu, Yan Xie, Shucai Zhang

**Affiliations:** 1 State Key Laboratory of Chemical Safety, Qingdao 266104, China; lizx.qday@sinopec.com (Z.L.); zhangfy.qday@sinopec.com (F.Z.); zhaosl.qday@sinopec.com (S.Z.); sunmb.qday@sinopec.com (M.S.); liujr.qday@sinopec.com (J.L.); 2 SINOPEC Research Institute of Safety Engineering Co., Ltd., Qingdao 266104, China

**Keywords:** poly(ethylene terephthalate), biodegradation, DuraPETase, sit-directed immobilization, PET-binding ability

## Abstract

Plastic pollution caused by poly(ethylene terephthalate) (PET) highlights the urgent need for efficient biodegradation strategies. However, PET hydrolases such as DuraPETase typically exhibit limited substrate affinity for PET and insufficient operational stability. Although conventional immobilization improves enzyme stability, it often compromises catalytic activity. Here, we design a PET-targeting, orientation-controlled immobilization strategy that overcomes this traditional trade-off and enables efficient PET biodegradation. Guided by rational structural analysis, three Cys variants (R53C, R59C, R224C) were engineered for site-specific covalent attachment to a PDA@SiO_2_ support with inherent PET-binding capability. The resulting conjugates (Dura_R53C_-PDA@SiO_2_, Dura_R59C_-PDA@SiO_2_, and Dura_R224C_-PDA@SiO_2_) displayed distinct catalytic and stability profiles. Among them, Dura_R59C_-PDA@SiO_2_ achieved the optimal balance between activity and stability, retaining kinetic properties comparable to the free enzyme and maintaining 87.6% residual activity after 2 h at 80 °C. Water contact angle measurements confirmed its PET-targeting behavior, as evidenced by the reduction in the PET contact angle from 85° to 45°. In 10-day degradation assays at 50 °C, Dura_R59C_-PDA@SiO_2_ released a total of 4865.32 μM degradation products, representing a 2.37-fold increase relative to free DuraPETase. These findings demonstrate an effective strategy for industrial enzymatic PET degradation and recycling.

## 1. Introduction

Plastics have become ubiquitous in modern society, serving essential functions in construction, packaging, textiles, and transportation. Among various types of plastics, poly(ethylene terephthalate) (PET) has gained particular attention because of its low cost, durability, and versatility [1,2]. Currently, the annual global production of PET reaches approximately 56 million tons, accounting for nearly 18.6% of the total plastic output worldwide [3]. However, most PET is utilized in single-use products, and nearly 95% of post-consumer PET ultimately enters the environment [4]. The intrinsic aromatic polyester backbone of PET confers exceptional chemical stability, making it highly resistant to degradation; complete decomposition is estimated to require over 100 years [5]. During this prolonged period, weathering processes fragment PET into micro- and nanoplastics with diameters smaller than 5 mm [6]. These particles can bioaccumulate in the human body through the food chain, leading to severe health risks such as gastrointestinal obstruction, endocrine disruption, and reproductive impairments [7]. Collectively, these findings underscore the urgent need for effective PET waste management.

Various strategies have been developed for managing PET waste, including landfilling, incineration, mechanical recycling, and chemical degradation [8]. However, nearly 60% of PET waste is still disposed of through landfilling. These conventional approaches suffer from limited efficiency and pose risks of secondary pollution. Given these shortcomings, there is an urgent need to explore alternative strategies that are both environmentally benign and resource-efficient. In this context, enzymatic degradation has emerged as a promising and sustainable solution. In 2016, Yoshida et al. identified a novel enzyme, PET hydrolase (PETase), capable of catalyzing the degradation of PET into bis(2-hydroxyethyl) terephthalate (BHET), mono(2-hydroxyethyl) terephthalate (MHET), terephthalic acid (TPA), and ethylene glycol (EG) [9]. Notably, TPA and EG are high-value monomers that can be directly reused for PET resynthesis or for the production of other polymeric materials, thereby enabling sustainable recycling of PET waste [10].

Enhancing the catalytic performance of PETase has recently become a central topic in the field of PET biodegradation. Cui et al. redesigned PETase and developed a mutant, DuraPETase, which exhibited significantly improved PET-degrading performance [11]. However, the industrial application of free DuraPETase remains challenging because of its limited stability under harsh processing conditions and its poor reusability [12]. To address these limitations, enzyme immobilization has been considered a promising strategy [13]. For example, PETase immobilized onto nanostructured Co_3_(PO_4_)_2_ via biomimetic mineralization (PETase@Co_3_(PO_4_)_2_) demonstrated markedly improved stability, including a broadened pH tolerance (6.0–10.0), an elevated optimum temperature (+10 °C), and sustained activity. The immobilized enzyme retained 75% of its initial activity after 12 days, whereas the free enzyme was almost completely inactivated [14]. These findings underscore the potential of enzyme immobilization to enhance catalytic performance for PET degradation.

Despite these advances, immobilization alone does not fully address the intrinsic challenges associated with PET biodegradation. Conventional random immobilization strategies may partially obstruct active sites or impose unfavorable orientations, thereby limiting substrate binding [15]. Moreover, the hydrophobic nature of PET surfaces impedes the effective enzyme-substrate interactions, constraining catalytic efficiency even when enzyme stability is improved [16,17]. Therefore, developing site-directed immobilization approaches that both preserve active-site accessibility and promote enzyme–PET interactions is essential for maximizing the catalytic potential of DuraPETase in PET degradation [18].

In this study, a polydopamine (PDA)-coated SiO_2_ carrier (PDA@SiO_2_) was prepared and employed for the site-directed immobilization of DuraPETase. The PDA coating serves two essential functions: first, its abundant reactive functional groups provide versatile sites for covalent conjugation of DuraPETase [19]; second, its inherent adhesive properties facilitate effective localization of the immobilized enzyme onto hydrophobic PET substrates [20]. To achieve site-directed immobilization, DuraPETase was rationally engineered by introducing Cys residues at defined positions, enabling specific covalent conjugation to PDA through thiol-catechol chemistry. The resulting conjugates were then comprehensively characterized in terms of their physicochemical properties, catalytic behavior, and PET degradation efficiency compared with the free enzyme. Overall, this study presents a rational strategy to enhance the stability and catalytic activity of DuraPETase, providing valuable insights for the development of enzyme-based strategies for sustainable PET recycling within a circular economy framework.

## 2. Results and Discussion

### 2.1. Screening of Mutation Sites for DuraPETase

Traditionally, the functional groups (-NH_2_ and -COOH) on the enzyme can serve as anchoring sites for their covalent immobilization [21,22]. Considering that the surface of wild-type DuraPETase contains abundant -NH_2_ and -COOH, the enzyme might be immobilized onto PDA@SiO_2_ surfaces in a non-specific, random orientation. To overcome this limitation and achieve site-directed immobilization, Cys (containing -SH) was considered; -SH exhibits markedly high reactivity with PDA via Michael addition or Schiff base reactions, thereby enabling rational control over the immobilization sites [23]. Accordingly, candidate residues were rationally screened for Cys substitution.

**Figure 1:**
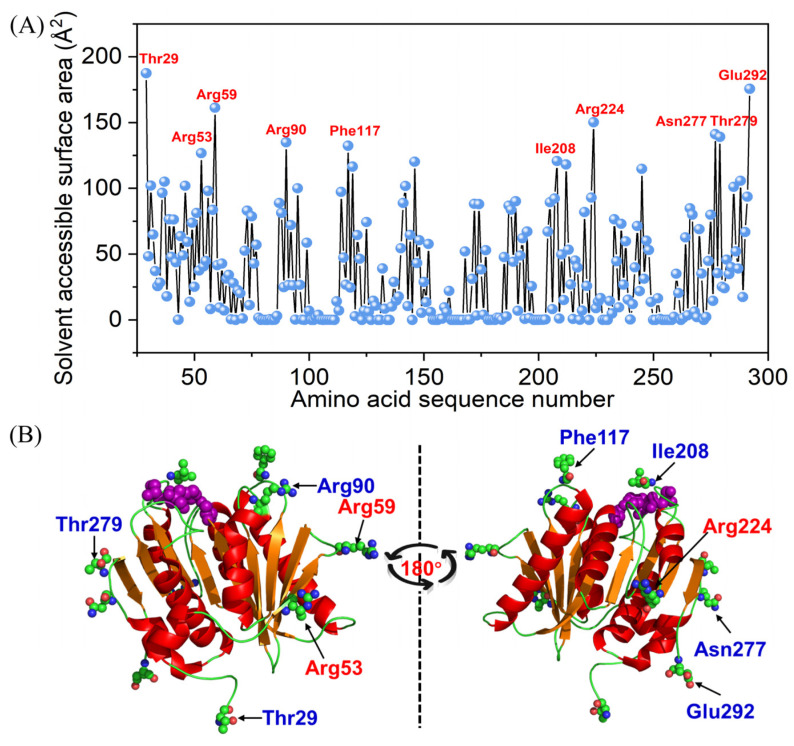
(**A**) The solvent-accessible surface area (SASA) of amino acids on DuraPETase. (**B**) Spatial distribution of the top ten amino acids ranked by SASA. The purple spheres indicate the active site of DuraPETase. The visualization was generated by Visual Molecular Dynamics (VMD 1.9.3), and the 3D structure of DuraPETase was obtained from the Protein Data Bank (PDB code: 6yk5).

As enzyme immobilization efficiency is largely governed by solvent accessibility of reactive groups, the SASA of all residues of DuraPETase was calculated using GETAREA, with the results presented in Figure 1A. The ten residues with the largest SASA values were Thr29 (187.5 Å^2^), Glu292 (175.6 Å^2^), Arg59 (161.2 Å^2^), Arg224 (150.2 Å^2^), Asn277 (141.2 Å^2^), Thr279 (139.1 Å^2^), Arg90 (134.92 Å^2^), Phe117 (132.32 Å^2^), Arg53 (126.64 Å^2^), and Ile208 (120.76 Å^2^). These residues were subsequently mapped onto the three-dimensional structure of DuraPETase (Figure 1B), where the catalytic triad (Ser160-His237-Asn206) is highlighted as purple spheres for reference [24]. To avoid potential loss of catalytic activity caused by mutations or subsequent immobilization, residues located near the active site were excluded (Phe117, Ile208, Arg90, Thr279, Asn277). These residues were considered high-risk, as modifications at these positions may compromise the integrity of the catalytic triad or introduce steric hindrance that obstructs substrate binding [25]. Accordingly, Thr29, Arg53, Arg59, Arg224, and Glu292 were retained as potential candidates for mutation. However, Thr29 and Glu292 are located at the N- and C-termini of DuraPETase, respectively, regions associated with structural flexibility and stability. Covalent immobilization at these positions may induce unpredictable conformational perturbations [26]; therefore, they were considered unsuitable for mutation.

Overall, Arg53, Arg59, and Arg224 emerged as favorable candidates. These residues possess high solvent accessibility and are positioned away from the catalytic center, minimizing potential disruption of the active site. Furthermore, the side chains of Arg are generally solvent-exposed and engage primarily in electrostatic or hydrogen-bonding interactions, rather than extensive intra-protein covalent networks [27]. Consequently, their substitution with Cys is unlikely to induce substantial conformational changes or compromise structural integrity [28]. Ultimately, Arg53, Arg59, and Arg224 were selected for mutation, yielding the variants Dura_R53C_, Dura_R59C_, and Dura_R224C_.

### 2.2. Preparation and Characterization of DuraPETase

**Figure 2:**
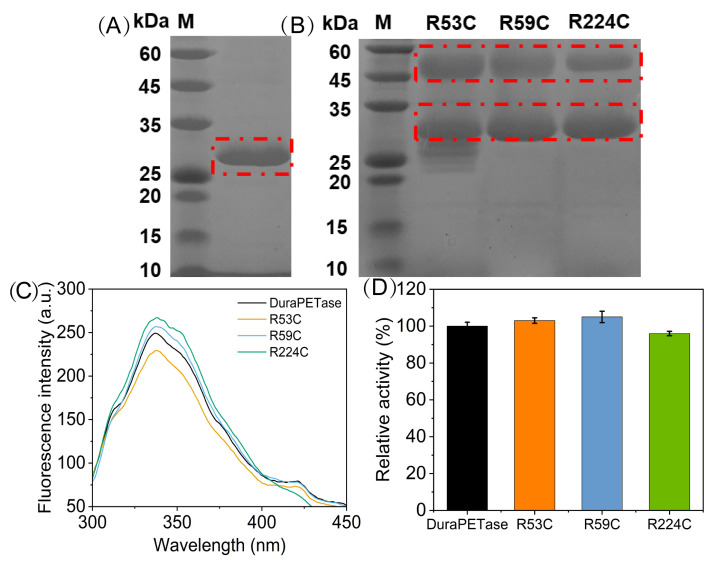
SDS-PAGE of (**A**) wild-type and (**B**) mutant DuraPETase. (**C**) Fluorescence spectra of DuraPETase and its variants. (**D**) Relative activity of wild-type and mutant DuraPETase. The activity of wild-type DuraPETase was defined as 100% for comparison.

The wild-type and variants were purified and further analyzed by (sodium dodecyl sulfate-polyacrylamide gel electrophoresis) SDS-PAGE. As shown in Figure 2A, a clear single band appeared in the range of 25–35 kDa, indicating that the wild-type DuraPETase (~28 kDa) was successfully prepared. No additional impurity bands were observed, indicating high homogeneity of the purified enzyme. For the three variants, SDS-PAGE analysis revealed two distinct protein bands in all samples (Figure 2B). In addition to the expected band at 28 kDa, corresponding to wild-type DuraPETase, additional bands were observed in the range of 45–60 kDa. These higher-molecular-weight proteins are likely attributed to dimer formation mediated by intermolecular disulfide bonds introduced by the Cys mutations.

Furthermore, the differences in tertiary structure between DuraPETase and its variants were investigated using intrinsic fluorescence spectroscopy. The fluorescence emission of proteins primarily originates from the aromatic residues Trp and Tyr. Alterations in their emission intensity or peak position can serve as sensitive indicators of conformational changes in the tertiary structure of the enzyme [24]. As shown in Figure 2C, the variants exhibit identical emission maxima (λ_max_ = 338 nm) compared with the wild-type DuraPETase. This indicates that the overall folding of DuraPETase was well preserved, regardless of the introduced mutations. The catalytic activities of the mutants were further evaluated to verify whether the structural consistency translated into functional retention. As shown in Figure 2D, R53C and R59C exhibited slightly enhanced relative activities of 103% and 105%, respectively, while R224C maintained 96% of the wild-type DuraPETase activity. Collectively, these results demonstrate that the selected variants (R53C, R59C, and R224C) were successfully designed and retain excellent catalytic performance, thereby providing reliable candidates for subsequent immobilization studies.

### 2.3. Characterization of the Carrier and Immobilized Enzyme

**Figure 3:**
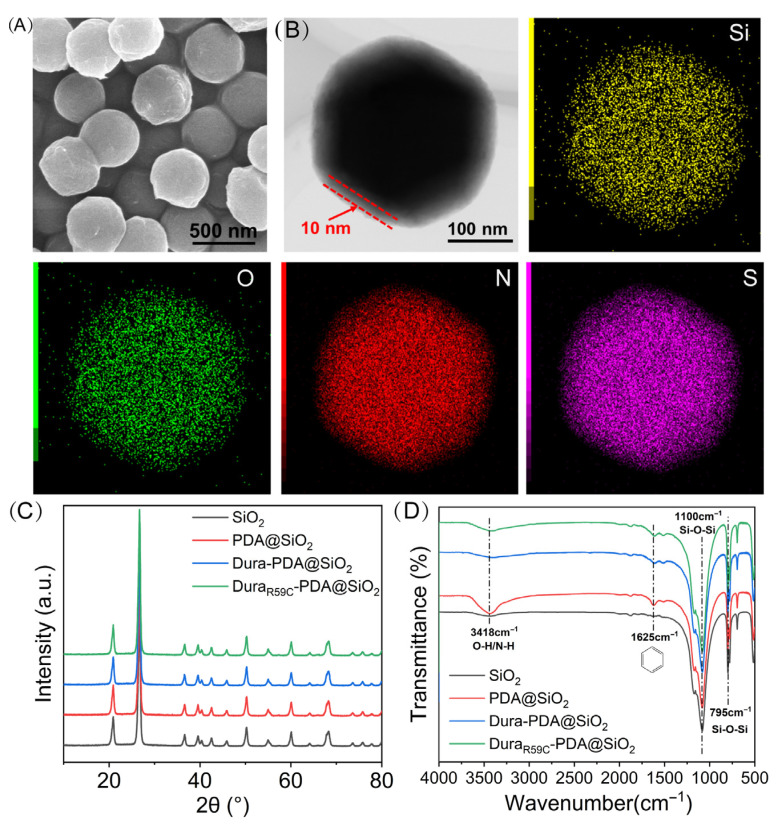
(**A**) SEM image of PDA@SiO_2_ nanoparticles. (**B**) TEM image and EDS analysis of Dura_R59C_-PDA@SiO_2_ nanoparticle. (**C**) XRD patterns of SiO_2_, PDA@SiO_2_, Dura-PDA@SiO_2_ and Dura_R59C_-PDA@SiO_2_. (**D**) FT-IR spectra of SiO_2_, PDA@SiO_2_, Dura-PDA@SiO_2_ and Dura_R59C_-PDA@SiO_2_.

As shown in Figure S1, the template SiO_2_ nanoparticles exhibited a smooth surface morphology with a uniform particle size distribution of approximately 300 nm. After dopamine treatment, their surface became slightly rougher, indicating the formation of the PDA layer (Figure 3A). Subsequently, the immobilized wild-type DuraPETase (Dura-PDA@SiO_2_) and the R59C variant (Dura_R59C_-PDA@SiO_2_) were selected as representative samples for further characterization.

As shown in Figure 3B, the TEM image of a representative DuraR59C-PDA@SiO_2_ nanoparticle reveals a distinct low-contrast shell surrounding the SiO_2_ core, corresponding to the deposited PDA layer with an estimated thickness of approximately 10 nm [29]. EDS analysis further corroborated the successful immobilization of the enzyme. The Si and O signals arise from the SiO_2_ template, while the detected N element may originate from the PDA layer or the immobilized enzyme. In contrast, the presence of S, an element uniquely derived from sulfur-containing amino acids in DuraR59C, provides definitive evidence that the immobilization strategy employed here successfully anchored the enzyme. Furthermore, the crystalline structure of the synthesized materials was further characterized. As shown in Figure 3C, the XRD pattern of all samples exhibits distinct diffraction peaks at approximately 20°, 26°, 36°, 39°, 50°, 60°, and 69°, which collectively match well with the characteristic reflections of crystalline SiO_2_ documented in the JCPDS card No. 46-1045 [30]. The presence of these peaks confirms that the SiO_2_ template retained its intrinsic crystallinity throughout PDA coating and subsequent enzyme immobilization processes. No additional diffraction signals attributable to PDA or the enzyme were observed, which is attributed to their predominantly amorphous nature. These results demonstrate that the immobilization process was mild and non-destructive, with the sample preparation following the intended technical protocol. Furthermore, FT-IR spectroscopy was conducted to elucidate the chemical changes occurring during the immobilization process. This technique enables the identification of characteristic functional groups and provides insight into potential interactions among the SiO_2_ template, the PDA layer, and the immobilized enzyme. As shown in Figure 3D, there are clear differences between the PDA/SiO_2_ and SiO_2_. In the unmodified SiO_2_ sample, the absorption peaks located at 795 cm^−1^ and 1100 cm^−1^ correspond to the symmetric and asymmetric stretching vibrations of Si-O-Si, respectively [31]. Following PDA deposition, additional functional groups are introduced. The pronounced enhancement of the broad band near 3418 cm^−1^ indicates the introduction of O-H and N-H, while the emergence of a distinct peak at 1625 cm^−1^, attributable to aromatic ring vibrations, provides definitive evidence of successful PDA coating [12]. After enzyme immobilization, a slight increase in transmittance around 3418 cm^−1^ is observed, likely due to partial coverage of PDA-derived O-H and N-H by immobilized enzyme molecules.

### 2.4. Catalytic Performance of the Immobilized Enzyme

**Figure 4:**
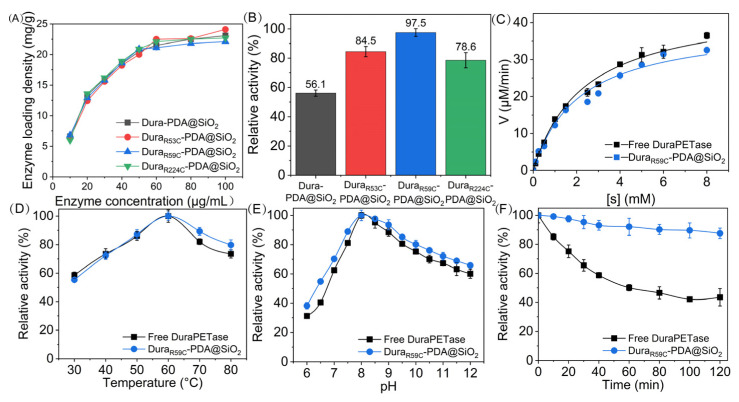
(**A**) Effect of initial enzyme concentration on the enzyme loading density. (**B**) The relative activity of four immobilized enzyme preparations. The activity of free DuraPETase was defined as 100% for comparison. (**C**) Kinetics of *p*-nitrophenyl acetate (*p*-NPA) hydrolysis catalyzed by free DuraPETase and Dura_R59C_-PDA@SiO_2_. Effects of (**D**) temperature and (**E**) pH on the relative activity of free and immobilized enzyme preparations. (**F**) Thermal stability of free and immobilized enzyme preparations at 80 °C for 2 h. The relative activity was defined as enzyme activity after incubation relative to initial activity.

The optimized immobilized condition for both the wild-type (Dura-PDA@SiO_2_) and mutant DuraPETase (Dura_R53C_-PDA@SiO_2_, Dura_R59C_-PDA@SiO_2_, and Dura_R224C_-PDA@SiO_2_) was first investigated. The enzyme loading density gradually increased as the enzyme concentration rose from 10 μg/mL to 60 μg/mL, after which it approached a plateau up to 100 μg/mL (Figure 4A). Therefore, an enzyme concentration of 60 μg/mL was identified as the most cost-effective condition for immobilization. Under this optimized concentration, the enzyme loading densities of Dura-PDA@SiO_2_, Dura_R53C_-PDA@SiO_2_, Dura_R59C_-PDA@SiO_2_, and Dura_R224C_-PDA@SiO_2_ were 21.5 mg/g, 22.5 mg/g, 21.1 mg/g, and 22.1 mg/g, respectively. In addition, the catalytic activities of the immobilized preparations were evaluated (Figure 4B). As compared to free DuraPETase, Dura-PDA@SiO_2_, Dura_R53C_-PDA@SiO_2_, Dura_R59C_-PDA@SiO_2_, and Dura_R224C_-PDA@SiO_2_ retained 56.1%, 84.5%, 97.5%, and 78.6% of the native activity, respectively. The significant activity loss of Dura-PDA@SiO_2_ indicates that non-specific immobilization can adversely affect enzyme performance. Such activity loss is commonly attributed to steric masking or partial obstruction of the catalytic cleft during immobilization, thereby limiting substrate accessibility [32]. In contrast, all three variants exhibited markedly higher activity retention after immobilization, underscoring the rationality of the mutation design in this study. To further interpret the differences among the mutants, the structural context was taken into consideration. R59C demonstrated the highest post-immobilization activity, which is likely due to its position within a flexible loop region, allowing the enzyme to retain conformational adaptability even when surface-bound [33]. By comparison, R53C and R224C are located on α-helical, where covalent anchoring may impose local conformational constraints or perturb tertiary structure, ultimately leading to reduced catalytic efficiency [34]. Taken together, subsequent investigations in this study focused on Dura_R59C_-PDA@SiO_2_.

To further elucidate the catalytic behavior of the immobilized system, the reaction kinetics of Dura_R59C_-PDA@SiO_2_ were evaluated and compared with those of the free enzyme (Figure 4C), and the kinetic parameters (*K*_m_ and *V*_max_) were subsequently calculated (Table 1). The *K*_m_ values of the free DuraPETase and Dura_R59C_-PDA@SiO_2_ were 2.33 mM and 2.12 mM, respectively, indicating that the orientation-controlled immobilization preserved the substrate-binding capacity of the DuraPETase. The slight decrease in *K*_m_ suggests an apparently enhanced affinity toward *p*-NPA, which may be attributed to the local enrichment of the small-molecule substrate by the PDA layer, given its known adsorption capability toward aromatic compounds [35]. The *k*_cat_ of Dura_R59C_-PDA@SiO_2_ (38.26 s^−1^) was slightly lower than that of the free enzyme (43.39 s^−1^), likely due to partial conformational constraints introduced during immobilization. Nevertheless, because the *K*_m_ value decreased marginally, the catalytic efficiency (*k*_cat_/*K*_m_) of the immobilized enzyme (18.03 s^−1^·mM^−1^) remained very close to that of the free enzyme (18.66 s^−1^·mM^−1^), indicating that the immobilization process did not compromise the overall catalytic performance of the enzyme.

Enzyme activity is typically sensitive to both temperature and pH. As shown in Figure 4D, the free enzyme and Dura_R59C_-PDA@SiO_2_ exhibited the same optimum temperature of 60 °C. This observation is reasonable, given that DuraPETase is a mutant derived from the wild-type enzyme and possesses increased structural rigidity, requiring relatively higher temperatures to achieve full catalytic activity [11]. Notably, the immobilized enzyme displayed superior thermal tolerance at elevated temperatures compared with its free counterpart, reflecting the stabilizing effect of immobilization on the enzyme’s conformational flexibility [36]. As shown in Figure 4E, both enzyme preparations exhibited an optimum pH of 8, which is suitable for the hydrolysis reaction catalyzed by DuraPETase [16]. Overall, these results suggest that the orientation-controlled immobilization did not substantially perturb the enzyme’s structural features or microenvironment, thereby preserving its intrinsic catalytic behavior [37].

Thermal stability is a critical determinant of the industrial applicability of enzyme preparations, as elevated temperatures often induce pronounced structural fluctuations that can lead to rapid deactivation. To evaluate the structural robustness, the thermal stabilities (at 80 °C) of the free enzyme and Dura_R59C_-PDA@SiO_2_ were systematically examined. As shown in Figure 4F, the free enzyme retained only 43.5% of its initial activity after incubation at 80 °C for 2 h, indicating substantial thermal denaturation. In contrast, the Dura_R59C_-PDA@SiO_2_ exhibited only a minor loss of activity under the same conditions and maintained 87.6% of its initial activity after 2 h. Together, these results demonstrate that immobilization effectively restricts detrimental conformational changes at elevated temperatures. Consequently, the prepared Dura_R59C_-PDA@SiO_2_ is well suited for long-term PET degradation.

### 2.5. Adhesion Behavior of Dura_R59C_-PDA@SiO_2_ onto PET Film

**Figure 5:**
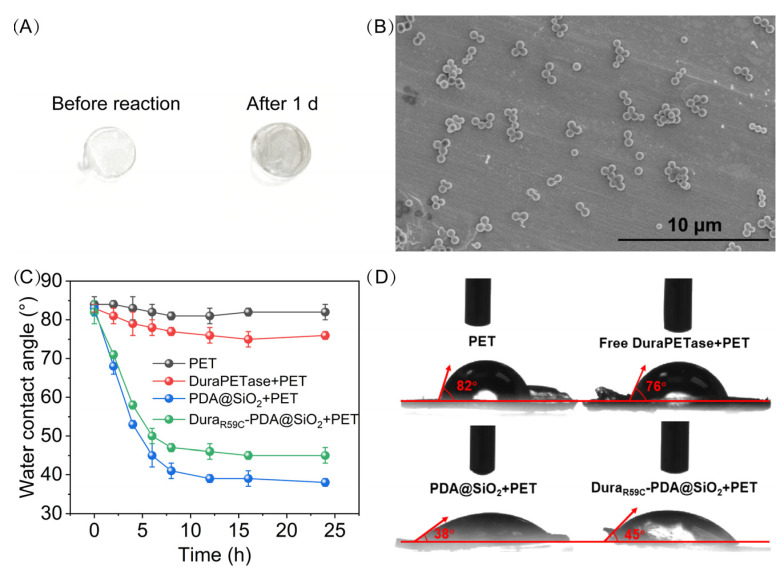
(**A**) Images of PET film before and after incubation with PDA@SiO_2_. (**B**) SEM image of PET film after 1 d incubation. (**C**) The change in water contact angle of PET films after 1 d incubation in different systems. (**D**) Water contact angle images of PET films after incubation in different systems.

Visual examination of the PET films before and after incubation (Figure 5A) revealed a pronounced darkening of the surface, indicating the deposition of substantial material. High-magnification SEM image (Figure 5B) further supports this finding, showing that the PET film was covered with nanoparticles matching those of PDA@SiO_2_ (Figure 3B).

Water contact angle measurements (Figure 5C) quantitatively characterize these interfacial alterations. Untreated PET film maintained a high water contact angle (~82°) over 24 h, reflecting its intrinsic hydrophobicity. Incubation with free DuraPETase yielded a modest reduction (to ~76°). This behavior can be attributed to the amphiphilic nature of the soluble enzyme, which confers a limited intrinsic affinity for the hydrophobic PET surface [20,38]. In contrast, exposure to the PDA@SiO_2_ carrier alone caused a substantial decrease in contact angle (to ~38°), indicating extensive surface modification and enhanced wettability. The immobilized enzyme preparation (Dura_R59C_-PDA@SiO_2_) led to an intermediate decrease (to ~45°), suggesting strong but somewhat attenuated surface adsorption ability relative to the PDA@SiO_2_ carrier. This reduction is likely attributable to the enzyme molecules partially masking or sterically hindering the surface functional groups of the PDA@SiO_2_ support that would interact more strongly with PET, thereby diminishing the overall adhesion efficiency [39].

Static contact angle images (Figure 5D) visually corroborate the final wettability differences. Collectively, these results demonstrate that orientation-controlled immobilization on PDA@SiO_2_ markedly enhances enzyme accumulation at the PET interface compared with free DuraPETase, thereby facilitating more efficient PET degradation.

### 2.6. PET-Biodegradation Ability of Dura_R59C_-PDA@SiO_2_

**Figure 6:**
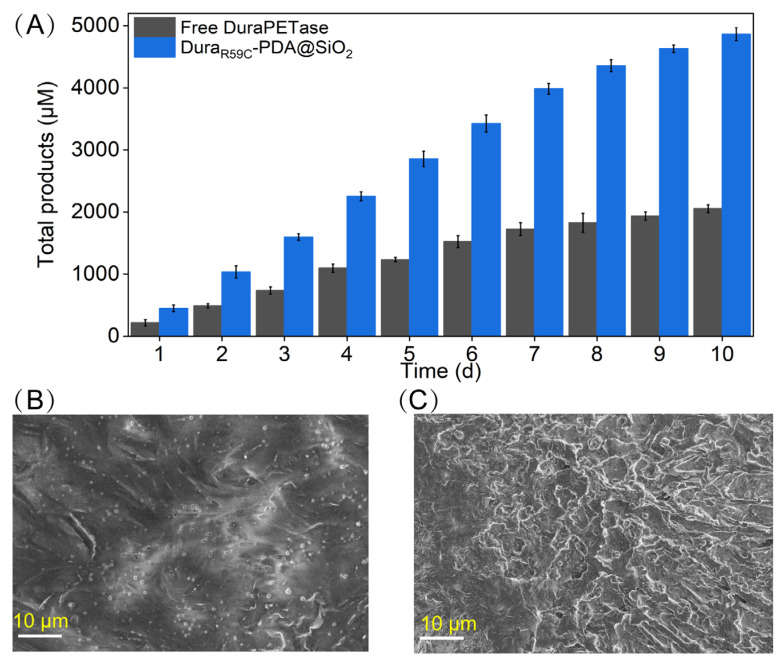
(**A**) Degradation of PET films by free DuraPETase and Dura_R59C_-PDA@SiO_2_. SEM images of PET films after treatment with (**B**) free DuraPETase and (**C**) Dura_R59C_-PDA@SiO_2_.

Long-term degradation experiments were conducted at 50 °C for 10 d to evaluate the catalytic performance of the Dura_R59C_-PDA@SiO_2_. As shown in Figure 6A, the total soluble degradation products (MHET and TPA) released by Dura_R59C_-PDA@SiO_2_ reached 4865.32 μM after 10 days, whereas the free-enzyme system produced only 2055.21 μM under identical conditions—corresponding to an approximate 2.37-fold enhancement in degradation efficiency. This improvement can be attributed, in part, to the markedly higher adhesion of the Dura_R59C_-PDA@SiO_2_ toward the PET surface (Figure 5). Stronger interfacial enrichment increases the local effective enzyme concentration at the PET interface and thus accelerates chain-end attack and ester-bond cleavage [40].

Furthermore, an analysis of the degradation kinetics provides additional mechanistic insight. In the free-enzyme system, the accumulation of PET hydrolysis products increased steadily during the first 6 d (~250 μM/d), but the rate of product formation declined gradually thereafter. This plateau behavior likely results from progressive conformational destabilization of DuraPETase at 50 °C, leading to partial unfolding or loss of catalytic competence over time. By contrast, the immobilized enzyme maintained a nearly linear increase in product formation throughout the 10 d period (~500 μM/d), with only a slight decrease in rate appearing around day 9. This late-stage slowdown might be related to substrate effects—namely, the preferential consumption of the more accessible amorphous PET segments during early degradation, leaving behind more recalcitrant crystalline regions that degrade more slowly [41].

Morphological characterization of the PET films (after 10 d reaction) further corroborated the above conclusions. The control PET film (incubation with reaction buffer) displayed the expected smooth and featureless surface, like the SEM image shown in Figure 5B. The PET film treated with free DuraPETase shows small and sparsely distributed pits (Figure 6B), consistent with the limited product. In sharp contrast, PET film treated by Dura_R59C_-PDA@SiO_2_ exhibited extensive surface roughening, with abundant cavities, trenches, and interconnected erosion channels (Figure 6C). This dramatic structural deterioration aligns with the higher product concentrations measured in solution (Figure 6A) and confirms that the immobilized enzyme induces a deeper and more continuous degradation process.

## 3. Materials and Methods

### 3.1. Chemical Reagents and Materials

SiO_2_ nanoparticles, dopamine hydrochloride (DA), *p*-NPA, and *p*-nitrophenol (*p*-NP) were purchased from Aladdin (Shanghai, China). Bovine serum albumin (BSA) was obtained from Sigma-Aldrich (St. Louis, MO, USA). Coomassie brilliant blue (CBB) G-250 was purchased from was from Alfa Aesar (Ward Hill, MA, USA). PET films were obtained from Goodfellow (Cambridge, UK). Isopropyl-beta-D-thiogalactopyranoside (IPTG), Na_2_HPO_4_, and imidazole were from Sangon Biotech (Shanghai, China). All other reagents were of analytical grade from Yuanli Chemical Technology (Tianjin, China).

### 3.2. Rational Screening for Mutation Sites

To identify suitable mutation sites for the site-directed immobilization of DuraPETase while minimizing impacts on catalytic activity, the SASA of all residues was calculated using GETAREA (http://curie.sdaponline.org/getarea.html (accessed on 27 August 2025)) [42]. The crystal structure of DuraPETase was obtained from the Protein Data Bank (PDB ID: 6YK5) [11]. SASA calculations were performed with a probe radius of 1.4 Å (corresponding to a water molecule), and both absolute and relative SASA values were computed for all non-hydrogen atoms. For each residue, the total SASA was normalized to its maximum theoretical exposure, and residues were ranked according to their relative SASA. The top ten residues with the highest relative SASA were initially selected as candidate mutation sites, since highly solvent-exposed residues are more accessible for covalent immobilization. To refine candidate selection, the spatial distribution of these residues was further examined using Visual Molecular Dynamics. Residues located within or near the substrate-binding cleft were excluded to avoid potential interference with enzymatic function [43]. Through this two-stage screening strategy, a final set of residues was identified for rational Cys substitution, enabling specific covalent conjugation with PDA in subsequent immobilization experiments.

### 3.3. Expression and Purification of Enzyme

The codon-optimized genes encoding wild-type and mutant DuraPETase were synthesized (Sangon Biotech, Beijing, China), cloned into the pET-22b (+) expression vector with a C-terminal His_6_-tag, and transformed into *Escherichia coli* BL21 (DE3). Protein expression and purification were performed according to previously reported procedures with minor modifications [15]. The recombinant strain was first cultured in 5 mL of Luria–Bertani (LB) medium at 37 °C for approximately 12 h. Subsequently, 3 mL of this seed culture was transferred into 300 mL of fresh LB medium and incubated at 37 °C with shaking at 160 rpm until the OD_600_ reached 0.6–0.8. Enzyme expression was induced by the addition of 0.5 mM IPTG, and cultivation proceeded at 16 °C for 24 h. Cells were then harvested by centrifugation at 5000 rpm for 30 min (4 °C). The collected cell was lysed, and the crude extract was purified using immobilized metal affinity chromatography (IMAC). The purified enzyme was subsequently concentrated into desalting buffer (50 mM Na_2_HPO_4_-HCl, 100 mM NaCl, pH 7.5) before analysis by SDS-PAGE. Protein concentration was determined using the Bradford assay with BSA as the standard.

### 3.4. Preparation of PDA@SiO_2_ Microsphere and Enzyme Immobilization

SiO_2_ nanoparticles with an average diameter of approximately 300 nm were used as templates for the preparation of PDA-coated SiO_2_ [12,30]. In detail, 300 mg of SiO_2_ were ultrasonically dispersed in 50 mL of Tris-HCl buffer (50 mM, pH 8.5). Subsequently, 100 mg of DA was added to the suspension, and the reaction was carried out under continuous magnetic stirring (1000 rpm) at 25 °C for 8 h. The resulting products were collected by centrifugation at 10,000 rpm for 15 min and washed six times with deionized water to remove unreacted residues. Finally, the black precipitates were freeze-dried under vacuum to yield PDA@SiO_2_ and stored at room temperature (~25 °C) for further use.

**Scheme 1:**
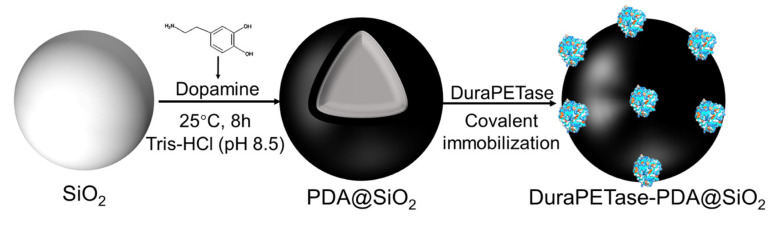
Synthetic process of PDA-coated support (PDA@SiO_2_) and immobilized enzyme.

Both wild-type and mutant DuraPETase were covalently immobilized onto the prepared PDA@SiO_2_ (Scheme 1). Briefly, 10 mg of PDA-coated SiO_2_ particles were dispersed in 10 mL of enzyme solution with a concentration ranging from 10 to 100 μg/mL. The mixture was incubated at 4 °C for 4 h to facilitate immobilization. The immobilized enzyme was collected by centrifugation at 10,000 rpm for 10 min and washed three times with the above-mentioned desalting buffer to remove any non-covalently adsorbed proteins. The supernatants collected during each step were analyzed to quantify the residual DuraPETase concentration, and the loading capacity was calculated by mass balance.

### 3.5. Characterization of Enzyme Performance

**Figure 7:**
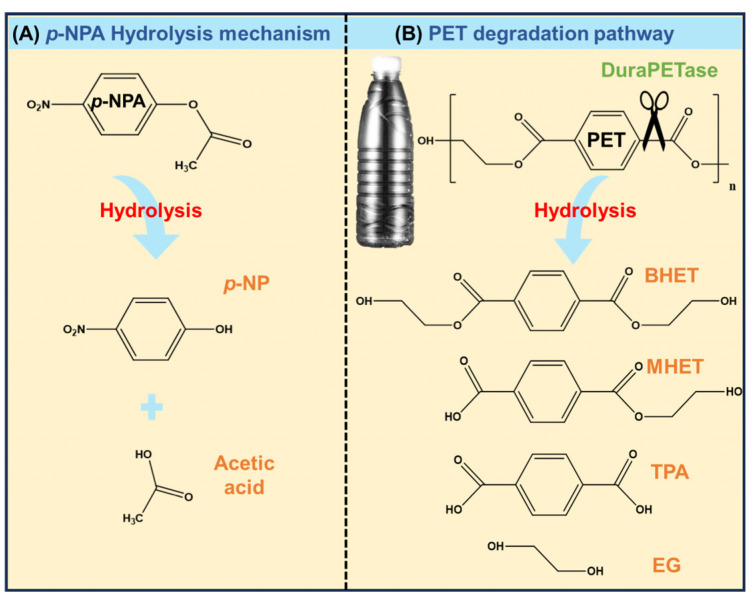
Schematic illustration of the hydrolysis mechanism of DuraPETase on (**A**) *p*-NPA and (**B**) PET.

As shown in Figure 7A, the catalytic activities of both free and immobilized enzyme preparations were determined using *p*-NPA as a model substrate. In a typical assay, 0.1 mL of enzyme solution (0.5 μg enzyme/mL) was mixed with 0.8 mL of reaction buffer (50 mM NaCl, 80 mM Na_2_HPO_4_-HCl, pH 7.0), followed by the addition of 0.1 mL of 80 mM *p*-NPA dissolved in acetonitrile. The reaction mixture was incubated at 30 °C for 5 min, after which the hydrolysis product, *p*-NP, was quantified by monitoring the absorbance at 412 nm. One unit (1 U) of enzyme activity was defined as the amount of enzyme required to release 1 μmol of *p*-NP per minute under the assay conditions.

To further characterize the enzymatic kinetics, initial reaction rates were measured using *p*-NPA concentrations ranging from 0.5 to 8 mM (30 °C, pH 7.0). The kinetic parameters were subsequently obtained by fitting the data to the Michaelis-Menten model,

(1) $$v=\frac{V_{\max}\left[S\right]}{K_{m}+[S]}$$

The reaction rate is expressed as *v* (μM/min), where [S] denotes the substrate concentration (mM), *V*_max_ represents the maximum reaction velocity (μM/min), and *K*_m_ corresponds to the Michaelis constant (mM).

To assess the influence of environmental factors on catalytic performance, the activities of free and immobilized DuraPETase were measured under a range of temperatures and pH values. Specifically, temperature-dependent activity profiles were obtained by assaying enzyme activity from 30 °C to 80 °C at pH 7.0, while pH-dependent activity was determined across a pH range of 6.0–11.0 at 30 °C. Herein, the maximum activity obtained under each condition was defined as 100%. Thermal stability was further evaluated by monitoring the residual activities of both enzyme preparations during incubation at 80 °C for 2 h, with the initial activity set as 100%.

### 3.6. Enzymatic Degradation of PET Film

PET films (6 mm in diameter, with a crystallinity of 8.1%) were prepared and sequentially pretreated with 1% SDS, distilled water, and absolute ethanol, followed by air-drying for subsequent experiments. To assess the catalytic activity of free and immobilized DuraPETase, the films were incubated in 1 mL of 50 mM glycine-NaOH buffer (pH 9.0) containing either free DuraPETase or immobilized DuraPETase (50 μg/mL). The reaction mixtures were incubated in a water bath (50 °C) for 10 d, with aliquots withdrawn at predetermined intervals for analysis. For product analysis, samples were immediately quenched by adding 160 mM phosphate buffer containing 20% DMSO, followed by heat inactivation at 85 °C for 10 min [10]. The heat-treated samples were then centrifuged, and the resulting supernatants were collected and analyzed via high-performance liquid chromatography (HPLC) according to previously reported protocols [15]. The concentrations of TPA and MHET generated during PET degradation (Figure 7B) were quantified, and their total amounts were summarized to represent the degradation efficiency [9,13].

### 3.7. Characterization of Materials

The physicochemical properties of the materials were systematically characterized using multiple analytical techniques. The morphology of the nanoparticle was examined by transmission electron microscopy (TEM, JEM-2100 F, JEOL, Tokyo, Japan), while surface features of the nanoparticle and PET films were assessed via scanning electron microscopy (SEM, Apreo S LoVac, FEI, Hillsboro, OR, USA). Crystalline structures were analyzed by X-ray diffraction (XRD, D8 Advance, Bruker, Bremen, Germany) over a 2θ range of 0–80° using Cu K_α_ radiation. The absorbance of the reaction mixtures at 412 nm was measured using a Lambda 25 spectrometer (PerkinElmer, Shelton, CT, USA). To evaluate interactions between PDA-modified materials and PET film, water contact angle measurements were conducted using a contact angle meter (Krüss, DSA-100, Hamburg, Germany). Briefly, PET films were separately incubated with free DuraPETase (50 μg/mL), PDA@SiO_2_, or immobilized DuraPETase under identical conditions. At predetermined intervals, droplets were deposited on the PET surface, and contact angles were continuously recorded to monitor changes in surface wettability over time.

## 4. Conclusions

In this study, we developed a novel immobilization strategy that improves both the stability and catalytic performance of DuraPETase. By introducing Cys mutations at rationally selected positions (R59C), DuraPETase was site-specifically immobilized on PDA@SiO_2_, a support that can adsorb onto PET surfaces. This design allowed the DuraPETase to be immobilized in a favorable orientation while maintaining access to its active site. The resulting Dura_R59C_-PDA@SiO_2_ retained kinetic properties close to those of the free enzyme and showed greatly improved thermal stability, with 87.6% residual activity after 2 h at 80 °C. Further characterizations supported the feasibility of our proposed strategy. SEM imaging showed a clear accumulation of Dura_R59C_-PDA@SiO_2_ on the PET surface during reaction. Water contact angle measurements further supported this observation: during reaction, the contact angle of the PET film decreased from 85° to 45°, indicating increased interaction between Dura_R59C_-PDA@SiO_2_ and the PET film. This interfacial enrichment, combined with the improved enzyme stability, enabled Dura_R59C_-PDA@SiO_2_ to degrade PET at a rate approximately 2.37 times higher than that of the free enzyme. The strategy described here provides a practical and scalable route to developing robust biocatalysts for PET depolymerization, offering valuable guidance for future enzyme-based plastic recycling technologies.

## Figures and Tables

**Table 1:** Kinetics Parameters of Free DuraPETase and Dura_R59C_-PDA@SiO_2_.

	Free DuraPETase	Dura_R59C_-PDA@SiO_2_
*V*_max_ (μM/min)	44.96	39.65
*k*_cat_ (s^−1^)	43.39	38.26
*K*_m_ (mM)	2.33	2.12
*k*_cat_/*K*_m_ (s^−1^·mM^−1^)	18.66	18.03

## Data Availability

The data presented in this study are available on request from the corresponding author.

## Supplementary Materials

The following supporting information can be downloaded at: https://www.mdpi.com/article/10.3390/molecules31101675/s1. Figure S1: SEM image of the pristine SiO_2_.
