# Supplementary material for: Site-Directed Immobilization of DuraPETase onto PET-Binding PDA@SiO2 for High-Efficiency PET Degradation
Source: Molecules. 2026 May 15;31(10):1675. doi: 10.3390/molecules31101675 (PMC13210071; doi:10.3390/molecules31101675)
Supplement: Supplementary file 1 [file molecules-31-01675-s001.zip › molecules-4278485-supplementary.pdf]

Site-directed immobilization of DuraPETase onto PET-binding PDA@SiO_2 for high-efficiency PET degradation

Zixuan Li [1,2], Fengyuan Zhang [1,2], Shaolei Zhao [1,2], Mingbo Sun [1,2], Jingru Liu [1,2], Yan Xie [1,2,*] and Shucai Zhang [1,2,*]

[1] *State Key Laboratory of Chemical Safety, Qingdao 266104, Shandong, China*

[2] *SINOPEC Research Institute of Safety Engineering Co., Ltd., Qingdao 266104, Shandong, China*

* Correspondence: zhangsc.qday@sinopec.com; Tel.: +86-532-83786653

xiey.qday@sinopec.com; Tel.: +86-532-83786071

1. Supporting Figure

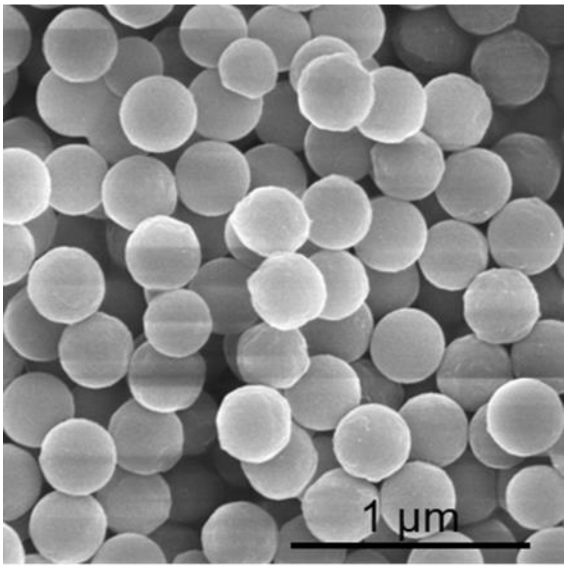

Figure S1. SEM image of the pristine SiO_2.
